# AR2, a novel automatic muscle artifact reduction software method for ictal EEG interpretation: Validation and comparison of performance with commercially available software

**DOI:** 10.12688/f1000research.10569.2

**Published:** 2017-04-04

**Authors:** Shennan Aibel Weiss, Ali A Asadi-Pooya, Sitaram Vangala, Stephanie Moy, Dale H Wyeth, Iren Orosz, Michael Gibbs, Lara Schrader, Jason Lerner, Christopher K Cheng, Edward Chang, Rajsekar Rajaraman, Inna Keselman, Perdro Churchman, Christine Bower-Baca, Adam L Numis, Michael G Ho, Lekha Rao, Annapoorna Bhat, Joanna Suski, Marjan Asadollahi, Timothy Ambrose, Andres Fernandez, Maromi Nei, Christopher Skidmore, Scott Mintzer, Dawn S Eliashiv, Gary W Mathern, Marc R Nuwer, Michael Sperling, Jerome Engel Jr, John M Stern

**Affiliations:** 1 Department of Neurology, David Geffen School of Medicine, University of California Los Angeles, Los Angeles, USA; 2 Department of Neurology, Jefferson Comprehensive Epilepsy Center, Thomas Jefferson University, Philadelphia, USA; 3 Department of Medicine, Statistics Core, University of California Los Angeles, Los Angeles, USA; 4 Department of Radiology, David Geffen School of Medicine, University of California Los Angeles, Los Angeles, USA; 5 Departments of Neurosurgery, Psychiatry, and Biobehavioral Medicine, David Geffen School of Medicine, University of California Los Angeles, Los Angeles, USA

**Keywords:** scalp EEG, electroencephalogram, muscle artifact, independent component analysis, seizure

## Abstract

*Objective:* To develop a novel software method (AR2) for reducing muscle contamination of ictal scalp electroencephalogram (EEG), and validate this method on the basis of its performance in comparison to a commercially available software method (AR1) to accurately depict seizure-onset location.

*Methods:* A blinded investigation used 23 EEG recordings of seizures from 8 patients. Each recording was uninterpretable with digital filtering because of muscle artifact and processed using AR1 and AR2 and reviewed by 26 EEG specialists. EEG readers assessed seizure-onset time, lateralization, and region, and specified confidence for each determination. The two methods were validated on the basis of the number of readers able to render assignments, confidence, the intra-class correlation (ICC), and agreement with other clinical findings.

*Results:* Among the 23 seizures, two-thirds of the readers were able to delineate seizure-onset time in 10 of 23 using AR1, and 15 of 23 using AR2 (p<0.01). Fewer readers could lateralize seizure-onset (p<0.05). The confidence measures of the assignments were low (probable-unlikely), but increased using AR2 (p<0.05). The ICC for identifying the time of seizure-onset was 0.15 (95% confidence interval (CI), 0.11-0.18) using AR1 and 0.26 (95% CI 0.21-0.30) using AR2.  The EEG interpretations were often consistent with behavioral, neurophysiological, and neuro-radiological findings, with left sided assignments correct in 95.9% (CI 85.7-98.9%, n=4) of cases using AR2, and 91.9% (77.0-97.5%) (n=4) of cases using AR1.

*Conclusions:* EEG artifact reduction methods for localizing seizure-onset does not result in high rates of interpretability, reader confidence, and inter-reader agreement. However, the assignments by groups of readers are often congruent with other clinical data. Utilization of the AR2 software method may improve the validity of ictal EEG artifact reduction.

## Introduction

The scalp electroencephalogram (EEG) is a critical diagnostic tool in the evaluation of seizures, but artifact from muscle contraction often limits its use because of the obscuring of the cerebrally generated potentials. This problem is present in 11% of ictal EEGs overall and up to 70% of frontal lobe seizures ^1– 3^. The inability, or lack of precision, to discern the seizure-onset zone from scalp EEG often necessitates additional testing, including (positron emission tomography) PET, magnetoencephalography, ictal Single-photon emission computed tomography (SPECT), and intracranial EEG ^4^. Each of these tests adds undesired time and cost to the evaluation.

Digital filters are the common approach to maximizing the likelihood of identifying a seizure-onset zone from EEG with muscle artifact. This filtering reduces muscle artifact by attenuating all frequencies beyond a selected value ^5^, but it may impair the integrity of the EEG recording since brain-generated potentials may be in the same frequency band ^6, 7^. Recently, new technologies to reduce muscle artifact based on independent component analysis (ICA) ^8– 10^ have become available. ICA derives spatial features that can remove artifacts that have static scalp topographies and time courses of activity that are distinct from that of EEG sources. ICA artifact correction is necessarily imperfect and will remove some neurogenic components of the EEG as well. However, the degree of EEG distortion may be negligible and ICA has proven effective at removing EMG and ocular artifacts from EEG data recorded from normal individuals in laboratory settings ^11– 20^. Prior studies have demonstrated that ICA-based methods improve the interpretation of artifact-laden ictal EEG recordings; in these studies researchers manually performed the ICA analysis prior to performing the EEG interpretation ^15, 16^. Automatic artifact reduction using ICA ^17– 19^ has become commercially available and is included in the latest versions of popular EEG viewer software ^20^. Ictal scalp EEG recordings present extraordinary challenges to ICA artifact reduction algorithms because the number of EMG artifact sources increases.

Despite the utilization of these software products by neurologists around the globe, the clinical benefit has not been established. It is also unknown if the new approaches introduce confounding artifacts that may lead to erroneous interpretations.

The goal of this study was to assess the validity of a commercially available EEG artifact reduction tool (AR1) that uses different montages and within electrode analysis to identify artefactual independent components ^20^, and compare its validity to a novel automatic artifact reduction tool (AR2), which was developed at the University of California Los Angeles on the basis of inter-reader agreement, confidence, and congruence with other clinical findings.

## Methods

### Implementation

The custom software algorithm involved importing EEG scalp recordings as European Data Format (EDF) files in Matlab 8.4 (Mathworks, Natick, MA). Prior to performing ICA to remove muscle artifact, the algorithm first identified epochs of the scalp EEG record contaminated by muscle artifact and determined the electrodes that were suspected of having high recording impedance during that epoch. The purpose of these calculations was to exclude these electrodes from the ICA calculations.

The imported EEG was band pass filtered (16–70 Hz) using a 500th order finite impulse response filter, i.e. FIR1 in referential montage. We then calculated the normalized instantaneous amplitude of the band-pass filtered signal using a Hilbert transform. This signal was smoothed using moving averaging, and the algorithm identified the longest epoch in which the time series remained greater than one standard deviation. We next calculated the normalized mutual information (MI) ^21^ adjacency matrix across all scalp electrode contacts during the (16–70 Hz) band-pass filtered artifact epoch of greatest duration and assigned each scalp EEG electrode a single MI value derived from the maximum pairwise MI values in the adjacency matrix. We then determined if this maximum mutual information value exceeded a threshold value defined by visual inspection of the scalp EEG used in the experimental dataset, and if that electrode should be included in subsequent artifact reduction processing. If the recording lacked an artifact epoch, or all channels were excluded, artifact reduction was applied to the referential recordings from all recording electrodes.

The high pass filtered (>16 Hz) scalp EEG was then separated into consecutive 120-second trials (24,000 data points) and each trial was processed using CUDAICA ^22, 23^. A 120 second trial length was chosen to optimize processing time. The purpose of the ICA was to separate the (>16 Hz) seizure activity, from the (>16 Hz) muscle artifact. The 16 Hz cut-off for the filter was chosen to isolate the vast majority of the muscle artifact. Independent components that explained an amount of variance above a particular threshold were excluded from the signal. The threshold was selected on the basis of the values of the raw and normalized mixing matrix (i.e. inverse weight matrix) calculated in each of the ICA iterations. We assumed that the last myogenic component and first neurogenic component can be differentiated on the basis of the inverse weight matrix, which provides the spatial distribution of each component, and identifying the independent component that account for the most variance with a focal spatial topography ^17^ defined on the basis of exceeding a normalized threshold of two standard deviations in at least one electrode of the inverse weight matrix. This threshold was chosen on the basis of visual inspection of the EEG in the experimental dataset and resulting independent components.

The pruned EEG calculated for each 120 second trial of EEG (i.e iteration of CUDAICA) was concatenated, and subsequently the entire raw ictal EEG was low pass filtered (<16 Hz) using a 500th order symmetric digital FIR filter, and the resulting low pass filtered EEG was reconstituted by addition of the waveforms with the high pass (>16 Hz) filtered EEG, following the exclusion of the independent components suspected to represent muscle artifact. The reconstituted and modified ictal EEG was exported from Matlab format to EDF for subsequent visual analysis.

### Operation

All computations were carried out using compiled Matlab 8.4 custom scripts on a cluster of HP SL230s Gen 8 ES-2670 nodes with dual-eight-core 2.6 GHz Intel ES-2670 central processing units, 4 GB of memory per core, and NVIDIA Tesla graphics processing units. Minimal system requirements for operating AR2 include Matlab v8.4 or above, an Intel Xeon CPU, 2 GB of memory, a NVIDIA GPU, which is CUDA compatible, and CUDAICA. For scalp EEG files exported from Neuroworkbench (Nihon-Kohden, Irvine, CA, USA), executing the AR2 software method requires only inputting the file name of the EDF file of interest at the command line. For EDF files exported from other equipment manufacturers, full automation of the AR2 software method can be easily accomplished with simple modifications of the input parameters.

### Patients and sample selection

We tested AR2 retrospectively using 23 seizures from eight adult patients with suspected focal-onset seizures undergoing evaluation at the UCLA Seizure Disorder Center. The patients and seizures were selected by S.A.W, whom was not a reviewer, from a review of consecutive clinical neurophysiology case conference presentations between January 1, 2014 and December 1, 2015 and based on case conference consensus that the ictal EEG records were uninterpretable due to muscle artifact contamination when reviewed with conventional digital filtering. For each of these patients, between 1–4 uninterpretable seizures were selected for inclusion in the study on the basis of ** a lack of ECG, electrode, and salt bridge artifact by S.A.W. Clinical data for each patient included seizure semiology, inter-ictal epileptiform abnormality, unobscured findings and radiological reports from MRI, PET, SPECT scans. The EEG and clinical records were deidentified and research informed consent was not required. This study was approved under UCLA IRB#15-001481. The video EEGs were acquired using a EEG-1200 amplifier (Nihon-Kohden, Irvine, CA) at a sampling rate of 200 Hz, low frequency cut-off 0.08 Hz. Electrodes were placed according to the 10–20 international system with the additional anterotemporal electrodes at T1/T2. The duration of the exported EEG recording included the entire seizure and a 2–3 minute peri-ictal epoch.

### Muscle artifact removal

AR1 was the commercially available Persyst v12 artifact reduction software ^20^ (Persyst Development, San Diego, CA). The methods are proprietary. AR2 was developed by S.A.W and involved a two-step procedure consisting of a custom algorithm. EEG processed by AR2 was also interpreted using the Persyst v12 artifact reduction software.

### Performance measures of AR1 and AR2

The AR1 and AR2 processed data were reviewed in Persyst v12 without video by 26 neurologists with a specialization in EEG, 20 of whom were board certified. The readers were blinded to which records received AR1 or AR2, and each reader reviewed the 46 seizures in random. Following review of each ictal record, the reader completed a multiple choice questionnaire ( Supplementary File 1), which assessed ability to visualize seizure-onset (Y,N) lateralize seizure-onset (L,R,N), locate the region of ictal onset (anterior temporal, anterior frontal, mid-temporal, temporal-parietal-occipital, occipital, none), and self-identify confidence of interpretation on a 5 point scale [(5) entirely confident (4) somewhat sure (3) probable (2) not confident (1) unlikely i.e. slight probability] for each measure. When time of onset, laterality, or the seizure onset region was not assigned the confidence was taken as (0). Readers were not provided with a definition of seizure-onset.

### EEG analysis

During the interpretation of the ictal EEG processed by AR1 or AR2, no restrictions were placed on the use of Persyst v12 built in EEG filters (low-pass, high-pass, band-pass), or changes to montage. A comment in each recording was used to demarcate the time prior to the clinical seizure but not the EEG onset. The assessment was not time limited.

### Statistical analysis

Differences in EEG interpretation utilizing AR1 and AR2 were assessed using the paired student’s t-test and the McNemar test on paired nominal data. The Bonferroni-Holm method was used to correct for multiple comparisons. Agreement across readers (Y,N,L,R), using either AR1 or AR2, was calculated using the inter-class correlation coefficient (ICC). For these outcomes, missing values were imputed to be in between non-missing values, and were analyzed using cumulative logit mixed effects models, which capture this ordering in the values and accounts for the clustering of readings into patients, and seizures within patients. Agreement across readers for onset region was calculated using a Fleiss kappa and treating the missing values as a category of response. Errors are given as standard error of the mean (s.e.m), unless otherwise specified.

## Results

### Implementation of the AR2 method

**Figure 1.**
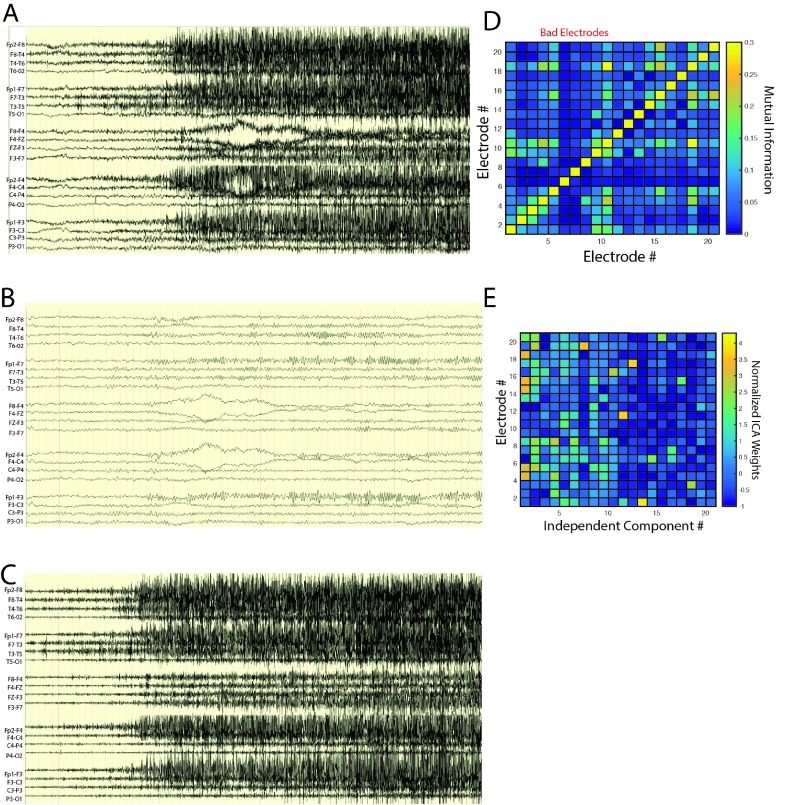
The AR2 method automatically separates independent components containing myogenic from neurogenic potentials. The AR2 method automatically separates independent components containing myogenic from neurogenic potentials in the beta and gamma band on the basis of spatial topography and explained variance. **A.** Unprocessed scalp ictal EEG recording that was deemed uninterpretable. **B.** The same epoch after applying a low pass (<16 Hz) filter demonstrating a lack of a convincing ictal rhythm. **C.** The ictal epoch after applying a high pass (> 16 Hz) filter demonstrating dense muscle artifact. **D.** An example of a mutual information adjacency matrix calculated during an epoch of artifact in the high pass (> 16 Hz) filtered scalp EEG recording. Three scalp electrode recordings exhibited relatively low mutual information with all other electrodes and were designated poor quality and excluded from further processing to optimize INFO-MAX ICA based artifact reduction. **E.** The normalized inverse weight matrix of all independent components across scalp electrode recordings for the seizure in panel A. Independent components 1-13 exhibited strong focality and were designated as containing myogenic potentials, while independent components 14 and above were designated neurogenic.

**Figure 2.**
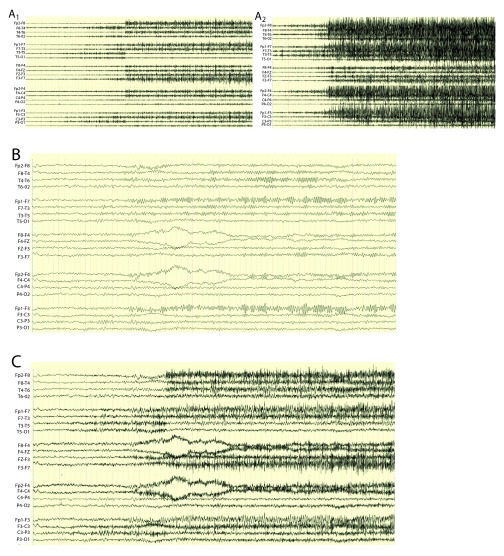
Ictal onset is revealed with reconstitution of the low pass (<16 Hz) ictal scalp EEG with the high pass (>16 Hz) neurogenic independent components. Reconstitution of the low pass (<16 Hz) ictal scalp EEG with the high pass (>16 Hz) neurogenic independent components reveals a clear ictal onset. **A.** The tentative neurogenic independent components ( **A1**) and myogenic independent components ( **A2**) derived from INFOMAX ICA processing of the high pass (> 16 Hz) filtered ictal scalp EEG recording are shown. The largest amplitude activity in the neurogenic components are evident frontally and in the left hemisphere. **B.** The low pass filtered ictal scalp EEG suggests a possible left frontal onset but a convincing ictal rhythm is lacking. **C.** Reconstitution of the low pass EEG with the neurogenic high pass (> 16 Hz) independent components results in an ictal EEG that demonstrates a more convincing left frontal onset consisting of beta-gamma oscillations with some clear phase reversals in F3 and F7.

**Figure 3.**
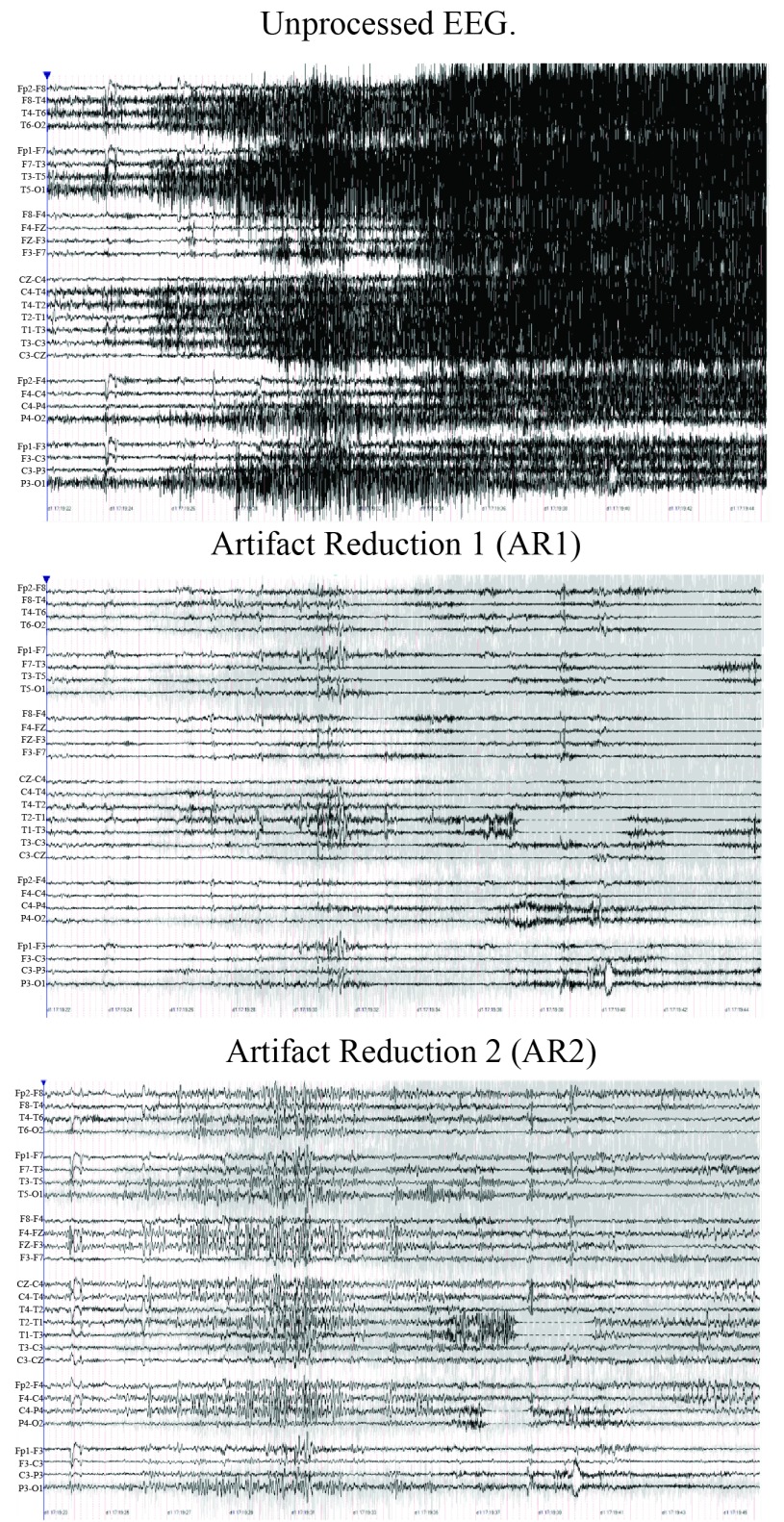
A comparison of the results of artifact reduction methodologies. Ictal scalp EEG recording from seizure 18 prior to artifact reduction processing (top), after processing with artifact reduction methodology 1 (AR1, middle), and after processing with artifact reduction methology 2 (AR2, bottom). Only processing with AR2 reveals a right hemispheric onset followed by clear spread to right frontal regions.

We applied the AR2 method developed at UCLA to the 23 seizures in the dataset. The method was automatic and unsupervised and separated the high-pass filtered (> 16 Hz) scalp EEG recordings into putative neurogenic and myogenic components ( Figure 1). After pruning the putative myogenic components, the putative neurogenic components were reconstituted with the low-pass filtered (< 16 Hz) scalp EEG ( Figure 2). The AR2 and AR1 processed scalp EEG recordings were subsequently inspected by the 26 specialists ( Figure 3).

### Identifying time of seizure-onset

**Figure 4.**
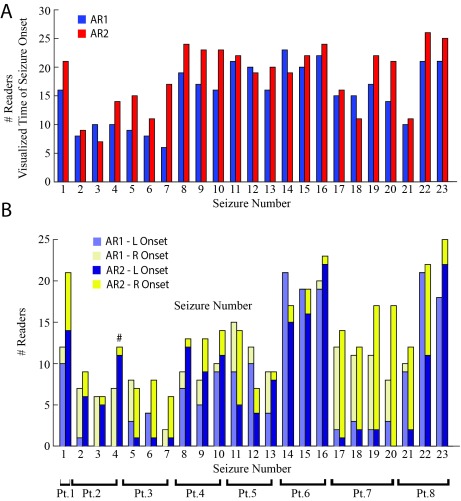
More readers could lateralize seizure onset utilizing AR2 as compared to AR1. More readers could visualize the time of seizure onset, and assign laterality to seizure onset utilizing AR2 as compared to AR1, and the assigned laterality of seizure onset sometimes differed between the two methods. **A.** Bar plot of the number of readers whom visualized the time of onset for each seizure utilizing AR1 (blue) or AR2 (red). Across seizures more readers visualized seizure onset utilizing AR2 compared with AR1 (p<0.01). Asterisks indicate statistically significant differences between the two methods in individual seizures (McNemar, p<0.05, Bonferroni-Holm corrected). **B.** Stacked bar plot of the number of readers selecting a left- or right-sided seizure onset utilizing AR1 (light blue, left; light yellow, right) or AR2 (dark blue, left; yellow, right). Across seizures more readers lateralized seizure onset utilizing AR2 compared with AR1 (p<0.01). Asterisks indicate statistically significant differences in individual seizures (McNemar, p<0.05, Bonferroni-Holm corrected), number sign indicates a significant change in the determination of laterality utilizing AR2 compared to AR1 (McNemar, p<0.05, Bonferroni-Holm corrected).

**Figure 5.**
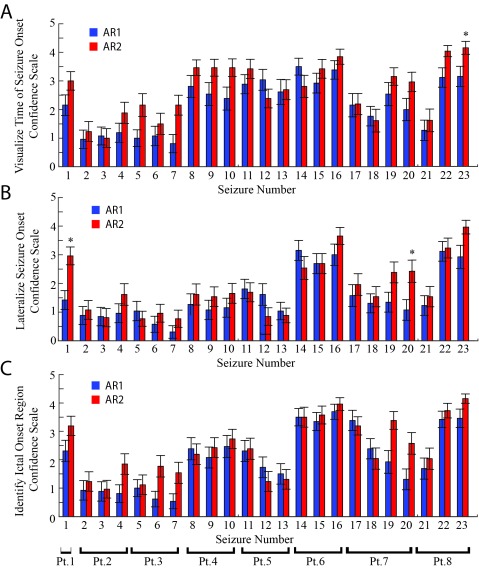
Confidence in the interpretation of ictal EEG onset improves with utilization of AR2 as compared to AR1. **A.** Bar plot of the mean confidence scale values for visualizing the time of seizure onset for the 23 seizures interpreted utilizing AR1 (blue), and AR2 (red). Across seizures, confidence scale values were greater when AR2 was utilized as compared with AR1 (p<0.01). Asterisks indicate differences in confidence values in individual seizures (p<0.05, Bonferroni-Holm corrected). Error bars are calculated as s.e.m. **B.** The respective mean confidence scale values for seizure onset lateralization. **C.** The respective mean confidence scale values for seizure focus localization. Across seizures, confidence scale values for lateralizing seizure onset, and identifying the seizure focus were greater when AR2 was utilized as compared with AR1 (p<0.05).

Across the 23 seizures considered previously uninterpretable with digital filtering ( Table 1) two-thirds of the readers were able to delineate the time of seizure-onset for 10 of the 23 using AR1, and 15 of the 23 using AR2 ( Figure 4A, n=23, paired t-test p<0.01, t=3.83). Across the 23 seizures, the mean confidence measure for the determination of seizure-onset was 2.68 +/- 0.19 (probable-not confident) when AR2 was utilized and 2.19 +/- 0.18 (not confident) with AR1 ( Figure 5A, d.f.=22, paired t-test, p<0.01, t=4.33). The inter-class coefficient (ICC) was 0.26 (95% Confidence Interval (CI) 0.21-0.30) with AR2, and 0.15 (95% CI 0.11-0.18) with AR1 (cumulative logit mixed effects models, p=0.333).

**Table 1.** Clinical description of patients. Clinical description of patients and ictal EEG laterality and focus assignments with AR1 and AR2. Abbreviations (L:left, R:right), PET findings refer to hypometabolism, SPECT findings to hyperperfusion. The focus was determined on a majority basis across all the assignments made by the readers for a subject’s seizure(s).

PatientAge Gender	Aura/Semiology	IEDs	Un- Obscured Seizure Onset Laterality	sMRI	PET/SPECT	Seizure Onset or Spread Laterality (AR2)	Seizure Onset or Spread Laterality (AR1)	AR2 focus	AR1 focus
#1 46M	Somato-sensory (warmth)/arousal from sleep, hyperkinetic,	none	left frontal ictal rhythm	nonlesional	normal PET, SPECT left insula	1. 14/21 L	1. 17/19 L	ant/mid temporal	ant temporal
#2 32M	Somato-sensory (discomfort)/right facial grimacing, right leg elevation, breath holding	none	none	nonlesional	PET right temporal, SPECT bilateral frontal lobes	2. 6/10 L 3. 6/8 L 4. 13/16 L	2. 6/7 R 3. 6/6 R 4. 7/7 R	ant.Frontal	mid. Temporal
#3 23M	Tachycardia/arousal from sleep, hyperkinetic, b/l dystonic posturing	none	none	nonlesional	normal PET	5. 8/10 R 6. 14/16R 7. 7/11 R	5. 6/11 L 6. 6/6 L 7. 6/6 R	frontal/mid temporal	mid temporal
#4 53M	Visual disturbance/ behavioral arrest, cursing, right arm dystonic posturing	L temporal	L anterior temporal	L MTS, L parietal encephalomalacia	PET L parietal	8. 18/19 L 9. 16/21L 10. 17/21 L	8. 14/17 L 9. 10/13 L 10. 21/23 L	ant/mid temporal	ant temporal
#5 20M	Vague/right head and eye version, right arm clonic movements,	L temporal	L temporal	L frontal polymicrogyria	normal PET	11. 11/18 R 12. 7/11 L 13. 9/10 L	11. 9/18 R 12. 10/13 L 13. 7/11 R	ant/mid temporal	ant/mid temporal
#6 27M	None/arousal from sleep, dyscognitive, right head and body version.	L frontal	L frontal	normal	PET L inferior frontal	14. 20/22 L 15. 21/24 L 16. 22/24 L	14. 25/25 L 15. 24/24 L 16. 24/25 L	ant frontal/ ant temporal	ant frontal/ ant temporal
#7 26F	None/nocturnal arousal or daytime events, hyperkinetic, right or left dystonic posturing	L and R temporal	None	Right middle cranial fossa arachnoid cyst	PET R parietal lobe	17. 21/23 R 18. 12/16 R 19. 21/23 R 20. 20/21 R	17. 20/23 R 18. 18/21 R 19. 14/16 R 20. 6/11 R	ant/mid temporal	ant/mid temporal
#8 19M	Lightheaded/loss of consciousness, right > left arm clonic movements, and posturing	L and R temporal	None	L mesial temporal CD, R>L gyrus rectus encephalomalacia	PET L>R temporal lobe	21. 12/16 R 22. 12/23 R 23. 23/26 L	21. 14/16 L 22. 23/24 L 23. 22/22 L	ant/mid temporal	ant/mid temporal

### Lateralizing and localizing seizure-onset

**Figure 6.**
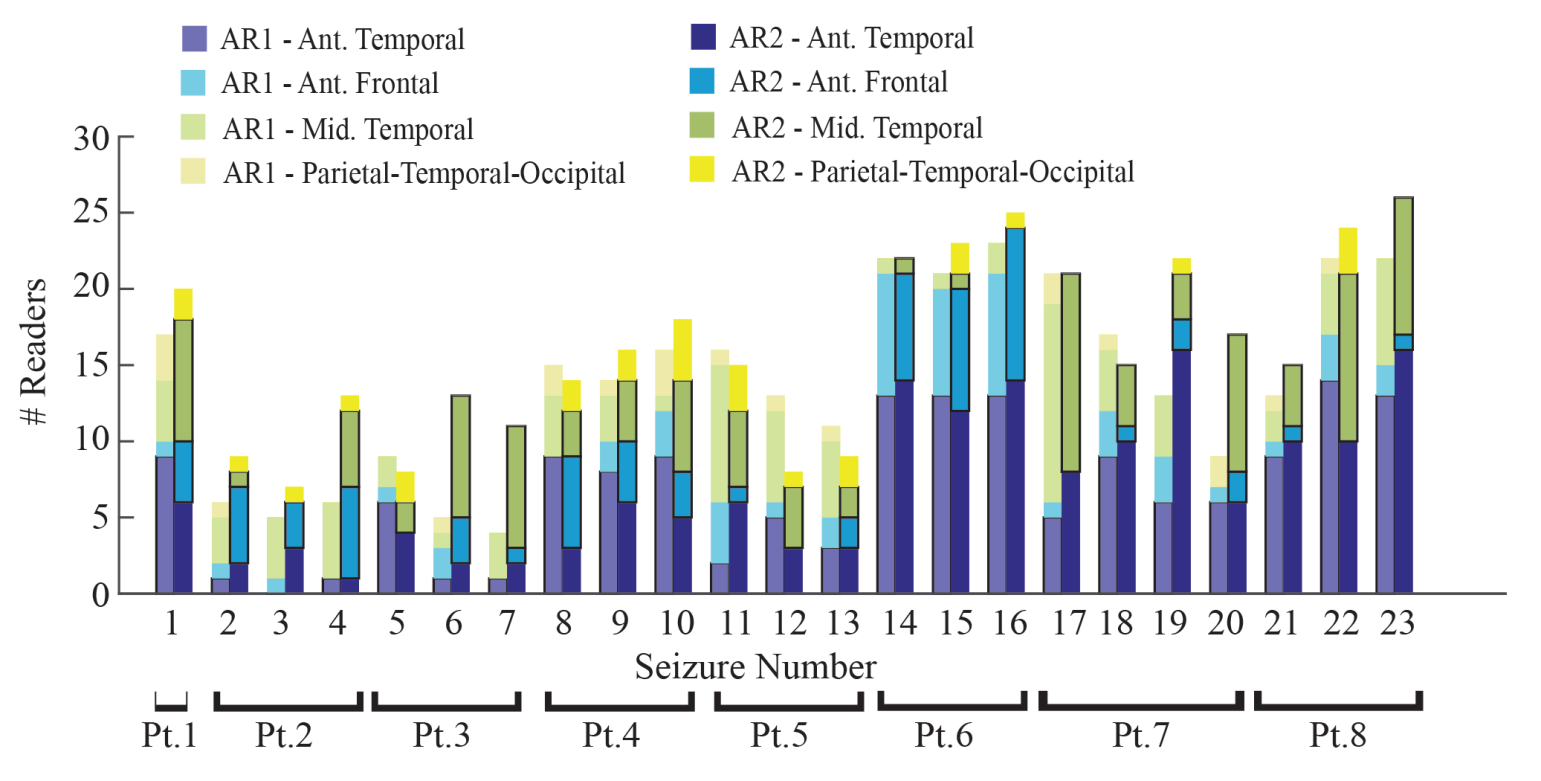
Differences in ictal onset region assignments using AR1 or AR2. Stacked bar plot of the ictal onset region assignments using either AR1 (lighter colors) or AR2 (darker colors) for all 23 seizures. Overall, across seizures, more readers were able to render an assignment using AR2 as compared to AR1 (p<0.05). Inter-reader agreement using for assigning the ictal onset region was marginal using either AR1 or AR2.

Compared with identifying the time of seizure-onset, fewer readers could lateralize seizure-onset after either AR1 or AR2 ( Figure 4B, d.f.=22, paired t-test, p<0.01, t=8.08 AR1, t=8.56 AR2). However, more readers were able to lateralize seizure-onset using AR2 compared to AR1 ( Figure 4B, d.f.=22, paired t-test, p<0.01, t=3.30) and readers were more confident with AR2, although both methods did not produce high levels of confidence. The mean confidence measure for seizure-onset lateralization was 1.87+/- 0.198 (not confident-unlikely) for AR2 and 1.54+/- 0.176 (not confident-unlikely) for AR1 ( Figure 5B, d.f.=22, paired t-test, p<0.01, t=2.85). The ICC was equivalent (cumulative logit mixed effects models, p=0.501) for AR1 (ICC=0.33 95% CI 0.30-0.37) and AR2 (ICC=0.28 95% CI 0.25-0.31). For localizing the region of seizure-onset reader confidence ( Figure 5C), and agreement was very low ( Figure 6, AR1 Fleiss’ kappa = 0.1199, 95% CI = 0.116-0.124, AR2 Fleiss’ kappa = 0.121, 95% CI =0.118-0.125). For one of the seizures, the laterality assignments were different when AR2 was used as compared to AR1 ( Figure 4B, McNemar p<0.05).

### Comparison of seizure-onset lateralization assignments with other clinical findings

We identified the patients with at least two consistent clinical findings that lateralized the suspected seizure-onset zone (SOZ). Compared to AR1, more readers were able to render seizure-onset laterality assignments using AR2, and these assignments were more often congruent with other clinical data ( Table 2). These clinical findings included seizure semiology, onset of seizures without EEG obscuration, structural MRI, PET, or SPECT findings. If any of the clinical findings were contradictory with respects to the laterality of the suspected SOZ, the SOZ was designated unknown. Overall, 4 patients (#1,4,5,6) had clinical findings that supported a left-hemispheric SOZ, and 1 patient (#7) had clinical findings that supported a right-hemispheric SOZ (Table S1). Among the 5 patients with clinical seizure onset lateralization based on independent data, if the reader lateralized the seizure-onset to the left using AR2 they were correct in 95.9% (95% CI 85.7-98.9%) of cases, but using AR1 they were correct in 91.9% (95% CI 77.0-97.5%) of cases ( Table 3, p<0.0607).

**Table 2.** Contingency table of agreement between assigned seizure onset laterality and other clinical findings. Contingency table of the agreement between seizure-onset laterality using AR1 (left), and AR2 (right) and the laterality of seizure-onset assigned on the basis of other clinical data for all the study patients and seizures. Note that clinical seizure-onset lateralization was not available for all patients, and when readers rendered a laterality decision that matched the laterality based on other clinical data, the assignments “agreed”.

		AR1						AR2		
		EEG seizure-onset lateralization						EEG seizure-onset lateralization		
		Y		N				Y		N
**Clinical seizure-** **onset** **lateralization**	**Y**	Agree 145	Disagree 32	187		**Clinical seizure-** **onset** **lateralization**	**Y**	Agree 171	Disagree 39	154
	**N**	83		151			**N**	107		127

**Table 3.** Agreement between seizure-onset laterality and other clinical findings. Agreement between seizure-onset laterality assignments using either AR1 or AR2 and the suspected laterality of the SOZ assigned on the basis of other clinical data. Parentheses indicate the 95% confidence interval. “n” refers to the number of subjects.

Artifact Reduction Method	Reader Assignment of Seizure-Onset Laterality	Percentage of reader assignments in concordance with SOZ laterality defined by other clinical criteria.
AR1	Right	59.3 (28.5-84.2) (n=1)
	Unknown	66.8 (38.1-86.9) (n=3)
	Left	91.9 (77.0-97.5) (n=4)
AR2	Right	61.8 (31.3-85.1) (n=1)
	Unknown	71.4 (42.8-89.3) (n=3)
	Left	95.9 (85.7-98.9) (n=4)

## Discussion

In this study, we present a new artifact reduction software, AR2, and its application compared with a commercially available tool, AR1. 26 neurologists used the two methods to interpret 23 ictal EEG recordings that were uninterpretable due to muscle artifact when reviewed with conventional filtering. The major findings from this study include: 1) the utilization of artifact reduction software results in non-uniform interpretation of ictal EEG, with many readers not able to render assignments; 2) when readers did render seizure-onset laterality assignments it often agreed with other clinical findings; 3) although the study size was small, the AR2 software method increased the number of readers that rendered assignments, and reader confidence suggesting it aids in diagnosis.

Both AR1 and AR2 are digital signal processing software tools ^8, 15, 20^ that may confound accurate ictal EEG interpretation by altering the appearance of the EEG. Digital filtering also can mislead ^5^. One concern about AR1 and AR2 relates to the uncertainty that myogenic activity was fully removed, and neurogenic components were unaffected during waveform alteration. Specifically, the readers were not confident in their interpretations, and the determination of seizure lateralization sometimes differed between the AR1 or AR2 methods. As such, the artifact reduction methods may introduce false positive findings. This demonstrates the limits of EEG artifact reduction approaches and puts the advantages into perspective.

The reliability of localization by ictal scalp EEG in the absence of artifact is between 65–75% for lateralization ^24^. Neurologists disagree more on the interpretation of ictal EEG processed with artifact reduction software, however the seizure-onset laterality assignments rendered by a quorum are often correct. Further refinement of this technology may successfully improve the efficiency of video-EEG monitoring and the utilization of epilepsy surgery; however, correlation with epilepsy resective surgery outcomes will be required for further validation.

With regard to AR2, the novel software method developed for this study, the slight improvement seen in ictal EEG interpretability after applying the method suggests that the algorithm can (1) sometimes produce signals that are, exclusively or mainly, EEG or EMG, and (2) identify which signals are of brain origin and which are contaminant. The effectiveness of AR2 could possibly be improved by utilizing autocorrelations to identify the myogenic independent components ^17^

One explanation for AR2’s ability to isolate myogenic from neurogenic activity may be related to the respective dipole generators of each. ICA produces independent components that may resemble single equivalent dipoles ^14^. Presumably, networks of myocytes exhibit shorter distance connectivity then networks of neurons that produce beta and gamma oscillations, and thus the two generators can be distinguished on the basis of the focality ^17^ of the independent components topography.

## Data and software availability

All software code for the new AR2 software developed by S.A.W. is openly and permanently available at https://github.com/shennanw/AR2.

Archived source code as at time of publication: doi, 10.5281/zenodo.229893 ^21^

License: GNU Public License 3.

The raw scalp ictal EEG files that were analyzed in this study using AR2, as well as the scalp ictal EEG files following processing using AR2 are available from Zenodo ^25^: **Dataset 1. Validity of two automatic artifact reduction software methods in ictal EEG interpretation.** Doi, 10.5281/zenodo.221095 ^22^ ( https://www.zenodo.org/record/221095#.WF63m7YrLdR)

The raw data used for the comparative assessments are available from Zenodo ^26^: **Dataset 2. Validity of two automatic artifact reduction software methods in ictal EEG interpretation.** Doi. 10.5281/zenodo.223329 ( https://zenodo.org/record/223329#.WHN-HLYrLdQ)

## Supplementary material

Supplementary File 1: Multiple choice questionnaire, completed by the reader after review of the ictal record.
