# Supplementary material for: AR2, a novel automatic muscle artifact reduction software method for ictal EEG interpretation: Validation and comparison of performance with commercially available software
Source: F1000Res. 2017 Apr 4;6:30. Originally published 2017 Jan 10. [Version 2] doi: 10.12688/f1000research.10569.2 (PMC5399961; doi:10.12688/f1000research.10569.2)
Supplement: Peer review history

# Peer review: Referee response for version 2

**DOI:** 10.5256/f1000research.12199.r21547

**Published:** 2017-04-12

**Authors:** Patrícia Figueiredo, Rodolfo Abreu

**Affiliations:** 1 Institute for Systems and Robotics and Department of Bioengineering, Instituto Superior Técnico University of Lisbon, Lisbon, Portugal

I appreciate the Authors' efforts to address most of my major concerns/suggestions. I approve this manuscript to pass peer review, however I believe it would still benefit from these minor corrections:

**Specific comments**

 4. " *ICA derives spatial features that can remove artifacts that have static scalp topographies and time courses of activity that are distinct from that of EEG sources." *I believe that what the Authors mean is "ICA derives spatial features that **can be used to identify** artifacts that...". Please confirm.

 5. I believe that the Authors forgot to include this relevant reference: *Urigüen, J.A., Garcia-Zapirain, B., 2015. EEG artifact removal—state-of-the-art and guidelines. J. Neural Eng. 12, 31001* ^1^. Please confirm.

 6. I feel that further details are still needed regarding AR1 because presently, it is not clear the rationale underlying AR1, nor its differences in relation to AR2.

We have read this submission. We believe that we have an appropriate level of expertise to confirm that it is of an acceptable scientific standard.

## References

[1] UrigüenJAGarcia-ZapirainB: EEG artifact removal-state-of-the-art and guidelines. *J Neural Eng*.2015;12(3) : 10.1088/1741-2560/12/3/031001 031001 10.1088/1741-2560/12/3/031001 25834104

# Peer review: Referee response for version 2

**DOI:** 10.5256/f1000research.12199.r21546

**Published:** 2017-04-07

**Authors:** David M. Groppe

**Affiliations:** 1 The Krembil Neuroscience Centre, Toronto, ON, Canada

The authors have provided a thoughtful revision that has address all my major suggestions/concerns. I think the current version of the manuscript is a positive contribution to the literature and approve it.

I have read this submission. I believe that I have an appropriate level of expertise to confirm that it is of an acceptable scientific standard.

# Peer review: Referee response for version 1

**DOI:** 10.5256/f1000research.11390.r20245

**Published:** 2017-03-16

**Authors:** David M. Groppe

**Affiliations:** 1 The Krembil Neuroscience Centre, Toronto, ON, Canada

In this manuscript, Weiss and colleagues present a novel algorithm for removing electromyographic (EMG) artifacts from ictal EEG recordings, called AR2. Moreover, they evaluate the performance of the algorithm on data from 8 patients and compare it to a similar commercial algorithm, AR1 (i.e., Persyst v12’s artifact correction software), using readings by 26 neurologists. The data chosen were so corrupted by EMG artifacts that they were not interpretable using conventional frequency-based filtering. Both AR1 and AR2 rely on independent components analysis (ICA) to remove EMG artifacts via spatial filters that are learned from the data. There is strong evidence that ICA is effective at removing EMG (and other EEG-artifacts) from data acquired in controlled, research settings[ref1] ^-^^2^. However, there may be too many EMG sources in highly polluted ictal recordings for ICA to work.

 In general, the authors found that both algorithms (1) made around 50% of the seizures interpretable with typically low levels of rater confidence and (2) produced very low-levels of inter-rater agreement. Nonetheless, when compelling seizure-onset lateralization was available from other sources of data (e.g., PET, SPECT), the algorithms led to EEG interpretations that were in concordance in about 80% of seizures (Table 2). Moreover, AR2 tended to slightly outperform AR1. Specifically, neurologists could interpret more seizures and tended to have more confidence in their interpretations following AR2 artifact correction. However, there was no statistically significant difference in inter-rater agreement between algorithms. The authors conclude from this that their AR2 algorithm “may improve the validity of ictal EEG artifact reduction.”

 In general, I think the authors’ work is laudable and that it is a valuable contribution to the literature. AR2 is well motivated given the evidence that ICA is successful at removing EMG (and other EEG artifacts) from data acquired in controlled research settings and the approach they have taken to validate their algorithm is generally sound. Moreover it is impressive that all of the seizures were read by a large number of neurologists, (26; although it is not clear how many were board certified in epilepsy or clinical neurophysiology) and that they have made all of their code and data public.

 However, there are some significant issues with this work that qualify their findings and should be addressed in revisions or future work:

 -As the authors note, the data for this study was obtained from a small number of patients (8, only 5 of whom had lateralized seizure foci based on independent data). Thus, it is not clear how robust some of their findings are (e.g., the small differences between AR1 and AR2 performance).

 -Although AR2 is a fully automatic algorithm, there are some arbitrary parameters of the algorithm (e.g., the mutual information threshold used to include an electrode in the artifact correction procedure) that must have been set based on exploratory analyses. If the data used to set these parameters are the same data used to validate the algorithm, then the authors are surely over-estimating, to some extent, the automatic performance of the algorithm. The authors need to specify what data were used to fix the parameters of AR2.

 -It is important to note that the authors chose extremely contaminated data to evaluate AR1 and AR2 and that these algorithms might be more useful when applied to less contaminated data.

 -If I understand the text correctly, AR2 excludes non-artifact contaminated electrodes from its analysis. You should include these electrodes in the ICA decompositions because they will help capture the neurogenic signal you are trying to preserve.

 -Since ICA necessarily removes some neurogenic signal along with EEG artifacts, it can help to quantify this by applying your algorithm to non-artifact polluted data ^2^. Adding such an analysis to these findings would help us to understand how and how much AR2 might be distorting EEG seizure activity. Electrodes closest to muscles are likely most affected.

 -For many statistical hypothesis tests the authors provide only p-values. It would be much more informative if the authors provided test statistics (e.g., t-scores, degrees of freedom), named the  type of test (e.g., cumulative logit mixed effect model) and confidence intervals. In particular, confidence intervals will be much better than p-values at communicating how important and robust these effects are ^3^.

 -Figures 4-5 report p<0.05 for the results of a large number of statistical tests (23 per subfigure) with no correction for multiple comparisons. You should perform some type of correction (e.g., Bonferroni-Holm or Benjamini & Hochberg’s false discovery rate control algorithm).

 -To interpret these results, it would greatly help to have inter-reader reliability and reader confidence values for non-artifact contaminated data. Can you get these from the existing literature?

 -I think the primary finding of this work is that neither AR1 nor AR2 provide robust artifact correction when applied to such heavily contaminated data and need to be improved. You should discuss what improvements (if any) you think could be made. For example, using higher-density EEG recordings could greatly help. With more electrodes, ICA’s performance should improve (given sufficient training data).

 In addition to those major points, here are some additional suggestions and points of consideration/clarification:

 The abstract should specify the consistency of AR1-derived lateralization with behavioural, neurophysiological, and neuro-radiological findings. Currently, only the consistency with AR2-derived lateralization is reported.

 -[pg 3]: Saying “ICA removes artifacts based on source-related features instead of frequencies.” is too vague to be informative. You might consider providing more details, such as “ICA derives spatial filters that can remove artifacts that have static scalp topographies and time courses of activity that are distinct from that of EEG sources. ICA artifact correction is necessarily imperfect and will remove some neurogenic components of the EEG as well ^4^. However, the degree of EEG distortion may be negligible and ICA has proven effective at removing EMG and ocular artifacts from EEG data recorded from neuronormal individuals in laboratory settings ^2^.”

 -The introduction should note why ICA might not be able to correct for EMG-ictal artifact, even though it has proven useful for less artifact-polluted research data. Specifically, it may fail because the number of EEG artifact sources may be much greater in ictal data.

 -You should include the article by De Vos et al. (2011) ^5^ in your review of previous algorithms for correcting EEG artifacts in clinical epilepsy data with ICA.

 -You say that EEG readings were provided by “26 neurologists with a specialization in EEG.” Please specify how many were board certified in epilepsy or clinical neurophysiology.

 -It appears that AR2 is applied to epochs that are not contaminated with EMG (pg 3, bottom left). Why try to correct artifacts that aren’t there?

 -Instead of saying “independent components of greatest order,” I think it is more conventional to say “independent components that account for the most variance.”

 -Please provide the specifications of the analog filter used to acquire the data.

 It would help to explicitly report the number of data points per electrode fed to ICA. The reliability of ICA is a function of this ^6^.

 -It might help to clearly state that the AR1 and AR2 processed data were both read using the same graphical user interface (i.e., Persyst’s). It took me a little while to figure this out and it’s great that you did this.

 -It would help to add titles to subfigures (if it is permitted by F1000’s formatting guidelines).

 -In Figure 1 there is no point to showing both the non-normalized ICA and normalized mixing matrix since the mixing matrix column scale is arbitrary. Just show the normalized mixing matrix. It would also help to view the mixing matrix weights as scalp topographies to see both the quality of the putative neurogenic and EMG ICs.

 -[pg 4] You say “Compared to AR1, more readers were able to render seizure-onset laterality assignments using AR2, and these assignments were more often congruent with other clinical data (Table 2).” However in Table 2, 82% of the seizures that were lateralizable with AR1 (i.e., 145/177) agree with clinical findings in contrast to 81% of seizures using AR2 (i.e., 171/210). I think percentage of agreement is more important than the number of seizures in agreement.

 -[pg 11] You say “Among the 8 patients, if the reader lateralized the seizure-onset to the left using AR2 they were correct in 95.9%….”. Do you mean “Among the 5 patients” with clinical seizure onset lateralization based on independent data?

 -I think your statement “With regard to AR2, the novel software method developed for this study, the slight improvement seen in ictal EEG interpretability after applying the method suggests that the algorithm can (1) reliably produce signals that are, exclusively or mainly, EEG or EMG, and (2) identify which signals are of brain origin and which are contaminant.” is overly strong. I think “sometimes” is more accurate than “reliably” given the low reader confidence and inter-reader agreement.

 -I don’t understand your statement “One explanation for AR2’s ability to isolate myogenic from neurogenic independent components may be that scalp EEG electrodes record weighted and summated far-field signals from all brain and muscle sources, as well as near-field electrode noise generated at the electrode/skin interface.” ICA can separate myogenic from neurogenic activity because they have distinct scalp topographies and largely independent time courses of activity.

 -It is fantastic that you have made both AR2’s code and your data publicly available. However, there is not enough documentation on your GitHub repo for me to be able to easily understand how to use it (what is scalp_input_matrix.mat?). A little bit more documentation would greatly help.

I have read this submission. I believe that I have an appropriate level of expertise to confirm that it is of an acceptable scientific standard, however I have significant reservations, as outlined above.

## References

[1] JungTPMakeigSHumphriesCLeeTWMcKeownMJIraguiVSejnowskiTJ: Removing electroencephalographic artifacts by blind source separation. *Psychophysiology*.2000;37(2) :163-78 10731767

[2] MognonAJovicichJBruzzoneLBuiattiM: ADJUST: An automatic EEG artifact detector based on the joint use of spatial and temporal features. *Psychophysiology*.2011;48(2) : 10.1111/j.1469-8986.2010.01061.x 229-40 10.1111/j.1469-8986.2010.01061.x 20636297

[3] GroppeDM: Combating the scientific decline effect with confidence (intervals). *Psychophysiology*.2017;54(1) : 10.1111/psyp.12616 139-145 10.1111/psyp.12616 28000255

[4] GroppeDMMakeigSKutasM: Independent component analysis of event-related potentials. *Cognitive Science Online*.2008;6(1) :11-44 Reference source

[5] De VosMDeburchgraeveWCherianPJMaticVSwarteRMGovaertPVisserGHVan HuffelS: Automated artifact removal as preprocessing refines neonatal seizure detection. *Clin Neurophysiol*.2011;122(12) : 10.1016/j.clinph.2011.04.026 2345-54 10.1016/j.clinph.2011.04.026 21705269

[6] GroppeDMMakeigSKutasM: Identifying reliable independent components via split-half comparisons. *Neuroimage*.2009;45(4) : 10.1016/j.neuroimage.2008.12.038 1199-211 10.1016/j.neuroimage.2008.12.038 19162199PMC3062525

# Response

**Published:** 2017-03-28

**Authors:** Shennan Weiss

**Affiliations:** Thomas Jefferson University

Dear Dr. David Groppe,

 We are grateful for your insightful and thoughtful comments and suggestions. Appended below are answers to your inquiries, and changes we have made to the manuscript.

 -As the authors note, the data for this study was obtained from a small number of patients (8, only 5 of whom had lateralized seizure foci based on independent data). Thus, it is not clear how robust some of their findings are (e.g., the small differences between AR1 and AR2 performance).

 -- The authors agree that this study is underpowered. Our findings are exploratory at best.

 -Although AR2 is a fully automatic algorithm, there are some arbitrary parameters of the algorithm (e.g., the mutual information threshold used to include an electrode in the artifact correction procedure) that must have been set based on exploratory analyses. If the data used to set these parameters are the same data used to validate the algorithm, then the authors are surely over-estimating, to some extent, the automatic performance of the algorithm. The authors need to specify what data were used to fix the parameters of AR2.

 -- You are correct that we used the experimental dataset to define the threshold values and thus we are likely over-estimating the performance of the algorithm. We clarify on (pg.3) and (pg.4) that the thresholds were defined using visual inspection of the experimental dataset in the revised manuscript.

 -It is important to note that the authors chose extremely contaminated data to evaluate AR1 and AR2 and that these algorithms might be more useful when applied to less contaminated data.

 -- On (pg.2) we now specify “Ictal scalp EEG recordings present extraordinary challenges to ICA artifact reduction algorithms because the number of EMG artifact sources increases.”

 -If I understand the text correctly, AR2 excludes non-artifact contaminated electrodes from its analysis. You should include these electrodes in the ICA decompositions because they will help capture the neurogenic signal you are trying to preserve.

 -- We apologize for the lack of clarity. We only excluded electrodes that had suspected increases in impedance. We specify on (pg.3) “Prior to performing ICA to remove muscle artifact, the algorithm first identified epochs of the scalp EEG record contaminated by muscle artifact and determined the electrodes that were suspected of having high recording impedance during that epoch. The purpose of these calculations was to exclude these electrodes from the ICA calculations.”

 -Since ICA necessarily removes some neurogenic signal along with EEG artifacts, it can help to quantify this by applying your algorithm to non-artifact polluted data ^2^. Adding such an analysis to these findings would help us to understand how and how much AR2 might be distorting EEG seizure activity. Electrodes closest to muscles are likely most affected.

 -- We agree that this analysis would be helpful and should be a focus of future study. Unfortunately, the EEG reviewers who participated in this study are not available to review non-ictal scalp EEG recordings.

 -For many statistical hypothesis tests the authors provide only p-values. It would be much more informative if the authors provided test statistics (e.g., t-scores, degrees of freedom), named the  type of test (e.g., cumulative logit mixed effect model) and confidence intervals. In particular, confidence intervals will be much better than p-values at communicating how important and robust these effects are ^3^.

 -- As you suggested we now provide t-scores, degrees of freedom, and have named the type of the test in the results. We provide confidence intervals for the cumulative logit mixed effects models results, and the correlation with other clinical data. S.E.M values are provided for the other comparisons in the figures included in the manuscript. The authors are in agreement the confidence intervals are essential to convey effect size ^3^ 

 -Figures 4-5 report p<0.05 for the results of a large number of statistical tests (23 per subfigure) with no correction for multiple comparisons. You should perform some type of correction (e.g., Bonferroni-Holm or Benjamini & Hochberg’s false discovery rate control algorithm).

 -- We have used your Matlab code to perform the Bonferroni-Holm correction on the p values obtained for the individual seizures. The results have been revised accordingly (see methods, statistical analysis).

 -To interpret these results, it would greatly help to have inter-reader reliability and reader confidence values for non-artifact contaminated data. Can you get these from the existing literature?

 -- We agree and the following sentence has been added to the discussion (pg. 13): The reliability of localization by ictal scalp EEG in the absence of artifact is between 65-75% for lateralization ^26^.

 -I think the primary finding of this work is that neither AR1 nor AR2 provide robust artifact correction when applied to such heavily contaminated data and need to be improved. You should discuss what improvements (if any) you think could be made. For example, using higher-density EEG recordings could greatly help. With more electrodes, ICA’s performance should improve (given sufficient training data).

 -- Thank you for this helpful suggestion, we have added the following sentence to the discussion (pg. 13): The effectiveness of AR2 could possibly be improved by utilizing autocorrelations to identify the myogenic independent components. We hope that this method can be optimized for 10/20 standard scalp EEG.

 In addition to those major points, here are some additional suggestions and points of consideration/clarification:

 The abstract should specify the consistency of AR1-derived lateralization with behavioural, neurophysiological, and neuro-radiological findings. Currently, only the consistency with AR2-derived lateralization is reported.

 -- We have provided the results for AR1 in the abstract as you suggested.

 -[pg 3]: Saying “ICA removes artifacts based on source-related features instead of frequencies.” is too vague to be informative. You might consider providing more details, such as “ICA derives spatial filters that can remove artifacts that have static scalp topographies and time courses of activity that are distinct from that of EEG sources. ICA artifact correction is necessarily imperfect and will remove some neurogenic components of the EEG as well ^4^. However, the degree of EEG distortion may be negligible and ICA has proven effective at removing EMG and ocular artifacts from EEG data recorded from neuronormal individuals in laboratory settings ^2^.”

 -- Thank you for your suggestion we have made these verbatim changes to the introduction (pg. 2)

 -The introduction should note why ICA might not be able to correct for EMG-ictal artifact, even though it has proven useful for less artifact-polluted research data. Specifically, it may fail because the number of EEG artifact sources may be much greater in ictal data.

 -- We have addressed this issue as mentioned in a prior comment to you.

 -You should include the article by De Vos et al. (2011) ^5^ in your review of previous algorithms for correcting EEG artifacts in clinical epilepsy data with ICA.

 -- done as suggested

 -You say that EEG readings were provided by “26 neurologists with a specialization in EEG.” Please specify how many were board certified in epilepsy or clinical neurophysiology.

 -- 20 of the readers were board certified as now specified on (pg.4.)

 -It appears that AR2 is applied to epochs that are not contaminated with EMG (pg 3, bottom left). Why try to correct artifacts that aren’t there?

 -- As specified in the methods we performed the ICA on 120 second trials irrespective of the beginning and end of the ictal EMG artifact. We used this approach in order to allow the algorithm to function in an automated and unsupervised manner.

 -Instead of saying “independent components of greatest order,” I think it is more conventional to say “independent components that account for the most variance.”

 -- We have made this modification as you suggested (pg. 3)

 -Please provide the specifications of the analog filter used to acquire the data. It would help to explicitly report the number of data points per electrode fed to ICA. The reliability of ICA is a function of this ^6^.

 -- We now specify 24,000 data points in the methods (pg.4)

 -It might help to clearly state that the AR1 and AR2 processed data were both read using the same graphical user interface (i.e., Persyst’s). It took me a little while to figure this out and it’s great that you did this.

 -- We have modified the methods as follows (pg. 5): The AR1 and AR2 processed data were reviewed in Persyst v12 without video by 26 neurologists with a specialization in EEG, 20 of whom were board certified.

 -It would help to add titles to subfigures (if it is permitted by F1000’s formatting guidelines).

 -- As far as I know this is not possible.

 -In Figure 1 there is no point to showing both the non-normalized ICA and normalized mixing matrix since the mixing matrix column scale is arbitrary. Just show the normalized mixing matrix. It would also help to view the mixing matrix weights as scalp topographies to see both the quality of the putative neurogenic and EMG ICs.

 -- We have changed the figure as you suggested and modified the legend.

 -[pg 4] You say “Compared to AR1, more readers were able to render seizure-onset laterality assignments using AR2, and these assignments were more often congruent with other clinical data (Table 2).” However in Table 2, 82% of the seizures that were lateralizable with AR1 (i.e., 145/177) agree with clinical findings in contrast to 81% of seizures using AR2 (i.e., 171/210). I think percentage of agreement is more important than the number of seizures in agreement.

 -- Thank you for this insightful point. The numbers do not refer to the number of seizures in agreement but rather to the number of observations i.e. assignments made that agreed with the laterality defined by other clinical data. Thus, more readers were able to render observations that agreed with other clinical data using AR2 as compared to AR1. However, as you point out the percentage of readers that made rendered a laterality decision that did not agree with the other clinical data using AR2 was comparable to AR1.

 -[pg 11] You say “Among the 8 patients, if the reader lateralized the seizure-onset to the left using AR2 they were correct in 95.9%….”. Do you mean “Among the 5 patients” with clinical seizure onset lateralization based on independent data?

 -- You are correct and we apologize for the lack of clarity. We have modified the results as follows (pg. 12): Among the 5 patients with clinical seizure onset lateralization based on independent data, …

 -I think your statement “With regard to AR2, the novel software method developed for this study, the slight improvement seen in ictal EEG interpretability after applying the method suggests that the algorithm can (1) reliably produce signals that are, exclusively or mainly, EEG or EMG, and (2) identify which signals are of brain origin and which are contaminant.” is overly strong. I think “sometimes” is more accurate than “reliably” given the low reader confidence and inter-reader agreement.

 -- We agree and have modified the sentence as you suggested (pg.13).

 -I don’t understand your statement “One explanation for AR2’s ability to isolate myogenic from neurogenic independent components may be that scalp EEG electrodes record weighted and summated far-field signals from all brain and muscle sources, as well as near-field electrode noise generated at the electrode/skin interface.” ICA can separate myogenic from neurogenic activity because they have distinct scalp topographies and largely independent time courses of activity.

 -- Thank you for pointing out that this sentence lacks clarity. We have modified this paragraph as follows (pg.13): One explanation for AR2’s ability to isolate myogenic from neurogenic activity may be related to the respective dipole generators of each. ICA produces independent components that may resemble single equivalent dipoles ^14^. Presumably, networks of myocytes exhibit shorter distance connectivity then networks of neurons that produce beta and gamma oscillations, and thus the two generators can be distinguished on the basis of the focality ^17^ of the independent components topography.  

 -It is fantastic that you have made both AR2’s code and your data publicly available. However, there is not enough documentation on your GitHub repo for me to be able to easily understand how to use it (what is scalp_input_matrix.mat?). A little bit more documentation would greatly help. 

 -- We are in the process of improving the documentation of AR2 on GitHub thank you for reviewing the source code.

**References**

 1. Jung TP, Makeig S, Humphries C, Lee TW, McKeown MJ, Iragui V, Sejnowski TJ: Removing electroencephalographic artifacts by blind source separation. *Psychophysiology*. 2000; **37** (2): 163-78 PubMed Abstract

 2. Mognon A, Jovicich J, Bruzzone L, Buiatti M: ADJUST: An automatic EEG artifact detector based on the joint use of spatial and temporal features. *Psychophysiology*. 2011; **48** (2): 229-40 PubMed Abstract | Publisher Full Text 

 3. Groppe DM: Combating the scientific decline effect with confidence (intervals). *Psychophysiology*. 2017; **54** (1): 139-145 PubMed Abstract | Publisher Full Text 

 4. Groppe DM, Makeig S, Kutas M: Independent component analysis of event-related potentials. *Cognitive Science Online*. 2008; **6** (1): 11-44 Reference Source 

 5. De Vos M, Deburchgraeve W, Cherian PJ, Matic V, Swarte RM, Govaert P, Visser GH, Van Huffel S: Automated artifact removal as preprocessing refines neonatal seizure detection. *Clin Neurophysiol*. 2011; **122** (12): 2345-54 PubMed Abstract | Publisher Full Text 

 6. Groppe DM, Makeig S, Kutas M: Identifying reliable independent components via split-half comparisons. *Neuroimage*. 2009; **45** (4): 1199-211 PubMed Abstract | Publisher Full Text

# Peer review: Referee response for version 1

**DOI:** 10.5256/f1000research.11390.r20461

**Published:** 2017-02-22

**Authors:** Patrícia Figueiredo, Rodolfo Abreu

**Affiliations:** 1 Institute for Systems and Robotics and Department of Bioengineering, Instituto Superior Técnico University of Lisbon, Lisbon, Portugal

Referee report

 The manuscript needs careful revision by a native English speaker within the scientific community. Although I feel that the performance measures used by the authors are adequate, and that a substantial number of EEG specialists quantified them, the overall results are poor, particularly in terms of the specialists’ confidence in their assessment. It would be therefore useful to compare the performance with additional methods, for instance, as to understand the behavior of the proposed performance measures across a larger set of methods.

 Specific Comments

**Title:**

 1. The title should mention that this novel method specifically addresses EEG artifacts induced by myogenic activity.

**Abstract:**

 2. (Results) The authors should include the consistency value also for AR1.

**Introduction:**

 3. (page 3, first paragraph) “ *Each of these tests adds undesired time and cost to the evaluation*”. I would say that the necessity of using additional imaging techniques depends on how precise one wants seizure-onset zone delineation to be, as scalp EEG has a poor spatial resolution and localization power. Please elaborate and/or re-phrase the sentence accordingly.

 4. (page 3, second paragraph) “ *ICA removes artifacts based on source-related features instead of frequencies”*. What do Authors mean with “source-related features”? Actually, there are several studies that use frequency-based criteria for the selection and subsequent removal of artifact-related sources…Please explain.

 5. (page 3, second paragraph) Please add more recent reviews/papers on the automatic IC selection for EEG cleaning, such as: *Chaumon, M., Bishop, D.V.M., Busch, N. a., 2015. A Practical Guide to the Selection of Independent Components of the Electroencephalogram for Artifact Correction. J. Neurosci. Methods. *or *Urigüen, J.A., Garcia-Zapirain, B., 2015. EEG artifact removal—state-of-the-art and guidelines. J. Neural Eng. 12, 31001*. ^1^

 6. (page 3, fourth paragraph) Authors refer to AR1 as a commercially available software, and in fact, detailed information about it is provided in reference [17]. However, the Authors should provide a brief description of the method because: 1) it is the only method which they compare their novel one with; and 2) so that future readers do not need to go through [17] in order to understand the overall rationale of AR1.

**Methods:**

 7. (page 3, *Implementation*, first paragraph) *“(…) a power spectral density algorithm to find extended intervals of elevated high frequency power across electrodes”*. The Authors provide no information about how this algorithm works, nor references; thus, it is presently not possible to reproduce this part of the study.

 8. (page 3, *Implementation*, first paragraph) The Authors need to justify their choices in general; particularly, why only compute the adjacency matrix between the epoch of greatest duration across all electrodes? Why compute the adjacency matrix in the first place, and not any other discriminative feature for the presence of muscle artifacts? Why only assign the maximum pairwise MI value in the adjacency matrix to a given electrode and ignore all the rest? How was the MI threshold determined?

 9. (page 3, *Implementation*, second paragraph) Again, the Authors need to provide more details overall. Why segment EEG into consecutive epochs of 120 s? How exactly was the variance threshold derived? Also, I did not understand why should be there any order associated with myogenic and neurogenic components (“ *We assumed that the last myogenic component and first neurogenic component (…)”*).

 10. (page 3, *Implementation*, second paragraph) I understand that one of the expected features of ICs reflecting muscle artifacts is having a focal spatial topography; however, bad channels are also reflected in ICs exhibiting this feature. Thus, I have severe concerns about false positives when using this criterion, as other myogenic-unrelated ICs are probably being selected as well, which may hinder a true assessment of the impact of muscle artifact correction.

 11. (page 3, *Implementation*, third paragraph) What does reconstitute mean in this context?

 12. (page 4, *Statistical analysis*) Since the performance of AR1 and AR2 is being assessed by 4 different performance measures obtained from 26 EEG specialists, it would be more accurate to use a 2-way repeated measures ANOVA (or its non-parametric equivalent, in the case of the samples not being normally distributed), followed by multiple comparison testing if necessary.

**Results:**

 13. The first three figures have very poor quality. In particular, it is nearly impossible to follow the overall (quite detailed) description of Figure 2 (and it is panel A _1_ on the top, left hand-side, and not A _2_). Also, the three panels in Figure 3 should be overlaid to facilitate the direct comparison between the two algorithms.

 14. Although AR2 outperforms AR1 for most of the performance measures, the results are still poor, making me wonder if either of these methods is suitable for EEG muscle artifact correction.

 Discussion

 15. (page 12, first paragraph) What do the Authors mean with “ *One concern about AR1 and AR2 relates to the lack of understanding of the waveform alteration”*?

 16. (page 12, fourth paragraph) “ *(…) (1) reliably produce signals that are, exclusively or mainly, EEG or MEG (…)”*. Please clarify and elaborate on this claim.

We have read this submission. We believe that we have an appropriate level of expertise to confirm that it is of an acceptable scientific standard, however we have significant reservations, as outlined above.

## References

[1] ChaumonMBishopDVBuschNA: A practical guide to the selection of independent components of the electroencephalogram for artifact correction. *J Neurosci Methods*.2015;250: 10.1016/j.jneumeth.2015.02.025 47-63 10.1016/j.jneumeth.2015.02.025 25791012

# Response

**Published:** 2017-03-28

**Authors:** Shennan Weiss

**Affiliations:** Thomas Jefferson University

Dear Dr. Figueiredo and Dr. Abreu,

 Thank you very much for your thoughtful and helpful comments and suggestions. We have substantially revised the manuscript according to your feedback as follows:

 1. The title should mention that this novel method specifically addresses EEG artifacts induced by myogenic activity.

 -- The title of the paper has been modified to “AR2, a novel automatic muscle artifact reduction software method for ictal EEG interpretation: Validation and comparison of performance with commercially available software”

**Abstract:**

 2. (Results) The authors should include the consistency value also for AR1.

 -- As you suggested we have added the consistency values for AR1 to the abstract.

**Introduction:**

 3. (page 3, first paragraph) “ *Each of these tests adds undesired time and cost to the evaluation*”. I would say that the necessity of using additional imaging techniques depends on how precise one wants seizure-onset zone delineation to be, as scalp EEG has a poor spatial resolution and localization power. Please elaborate and/or re-phrase the sentence accordingly.

 -- We have modified the introduction as follows (pg. 3): The inability, or lack of precision, to discern the seizure-onset zone from scalp EEG often necessitates additional testing, …

 4. (page 3, second paragraph) “ *ICA removes artifacts based on source-related features instead of frequencies”*. What do Authors mean with “source-related features”? Actually, there are several studies that use frequency-based criteria for the selection and subsequent removal of artifact-related sources…Please explain.

 -- Thank you for this instructive feedback. We have modified the introduction as follows (pg.3): ICA derives spatial features that can remove artifacts that have static scalp topographies and time courses of activity that are distinct from that of EEG sources.

 5. (page 3, second paragraph) Please add more recent reviews/papers on the automatic IC selection for EEG cleaning, such as: *Chaumon, M., Bishop, D.V.M., Busch, N. a., 2015. A Practical Guide to the Selection of Independent Components of the Electroencephalogram for Artifact Correction. J. Neurosci. Methods. *or *Urigüen, J.A., Garcia-Zapirain, B., 2015. EEG artifact removal—state-of-the-art and guidelines. J. Neural Eng. 12, 31001*. ^1^

 -- Thank you for suggesting the inclusion of this important methods article. We now cite this article in the introduction and discussion.

 6. (page 3, fourth paragraph) Authors refer to AR1 as a commercially available software, and in fact, detailed information about it is provided in reference [17]. However, the Authors should provide a brief description of the method because: 1) it is the only method which they compare their novel one with; and 2) so that future readers do not need to go through [17] in order to understand the overall rationale of AR1.

 -- Although the complete methods for AR1 are not included in reference 17 we have modified the introduction as follows (pg.3): The goal of this study was to assess the validity of a commercially available EEG artifact reduction tool (AR1) that uses different montages and within electrode analysis to identify artefactual independent components ^20^, and compare its validity to a novel automatic artifact reduction tool (AR2)…

**Methods:**

 7. (page 3, *Implementation*, first paragraph) *“(…) a power spectral density algorithm to find extended intervals of elevated high frequency power across electrodes”*. The Authors provide no information about how this algorithm works, nor references; thus, it is presently not possible to reproduce this part of the study.

 8. (page 3, *Implementation*, first paragraph) The Authors need to justify their choices in general; particularly, why only compute the adjacency matrix between the epoch of greatest duration across all electrodes? Why compute the adjacency matrix in the first place, and not any other discriminative feature for the presence of muscle artifacts? Why only assign the maximum pairwise MI value in the adjacency matrix to a given electrode and ignore all the rest? How was the MI threshold determined?

 -- We agree with your comments #7 and #8. We now specify in the methods that the reason we performed this analysis was (pg.3): “Prior to performing ICA to remove muscle artifact, the algorithm first identified epochs of the scalp EEG record contaminated by muscle artifact and determined the electrodes that were suspected of having high recording impedance during that epoch. The purpose of these calculations was to exclude these electrodes from the ICA calculations.”

 The method used to determine the artifact epoch had actually been modified prior to submission of version 1 of the manuscript. We now better describe this algorithm as “We then calculated the normalized instantaneous amplitude of the band-pass filtered signal using a Hilbert transform. This signal was smoothed using moving averaging, and the algorithm identified the longest epoch in which the time series remained greater than one standard deviation.”

 9. (page 3, *Implementation*, second paragraph) Again, the Authors need to provide more details overall. Why segment EEG into consecutive epochs of 120 s? How exactly was the variance threshold derived? Also, I did not understand why should be there any order associated with myogenic and neurogenic components (“ *We assumed that the last myogenic component and first neurogenic component (…)”*).

 -- We agree with your comment and apologize for the lack of clarity. We now specify that (pg.4): A 120 second trial length was chosen to optimize processing time.

 In addition, the method have been modified as follows (pg. 4): “We assumed that the last myogenic component and first neurogenic component can be differentiated on the basis of the inverse weight matrix, which provides the spatial distribution of each component, and identifying the independent component that account for the most variance with a focal spatial topography ^17^ defined on the basis of exceeding a normalized threshold of two standard deviations in at least one electrode of the inverse weight matrix. This threshold was chosen on the basis of visual inspection of the EEG in the experimental dataset and resulting independent components.”

 10. (page 3, *Implementation*, second paragraph) I understand that one of the expected features of ICs reflecting muscle artifacts is having a focal spatial topography; however, bad channels are also reflected in ICs exhibiting this feature. Thus, I have severe concerns about false positives when using this criterion, as other myogenic-unrelated ICs are probably being selected as well, which may hinder a true assessment of the impact of muscle artifact correction.

 -- We agree with your concerns however in the algorithm we already excluded bad channels using the algorithm described with reference to comments #7 and #8.

 11. (page 3, *Implementation*, third paragraph) What does reconstitute mean in this context?

 -- We now specify in implementation (pg.4) that: the resulting low pass filtered EEG was reconstituted by addition of the waveforms with the high pass (>16 Hz) filtered EEG

 12. (page 4, *Statistical analysis*) Since the performance of AR1 and AR2 is being assessed by 4 different performance measures obtained from 26 EEG specialists, it would be more accurate to use a 2-way repeated measures ANOVA (or its non-parametric equivalent, in the case of the samples not being normally distributed), followed by multiple comparison testing if necessary.

 -- We appreciate this helpful feedback. Dr. David Groppe the other reviewer of the manuscript suggested that we use paired t-tests and provide the t-value in order to convey effect size to the reader. We have followed his recommendations. Including both 2-way repeated measures ANOVA and paired t-tests would confuse the reader. 

**Results:**

 13. The first three figures have very poor quality. In particular, it is nearly impossible to follow the overall (quite detailed) description of Figure 2 (and it is panel A _1_ on the top, left hand-side, and not A _2_). Also, the three panels in Figure 3 should be overlaid to facilitate the direct comparison between the two algorithms.

 --

 a) We have made grammatical changes to figure 2, and corrected the figure A1 vs. A2 labeling. We apologize for this oversight.

 b) We attempted to overlay the panels of figure 3 but the result was confusing and not visually appealing. Therefore, we cannot provide this suggested change.

 14. Although AR2 outperforms AR1 for most of the performance measures, the results are still poor, making me wonder if either of these methods is suitable for EEG muscle artifact correction.

 -- We agree and point out in the discussion that the readers were not confident in their interpretations using either AR1 or AR2 in the discussion (pg. 13).

 Discussion

 15. (page 12, first paragraph) What do the Authors mean with “ *One concern about AR1 and AR2 relates to the lack of understanding of the waveform alteration”*?

 -- This sentence has been modified to provide more clarity (pg.13): “One concern about AR1 and AR2 relates to the uncertainty that myogenic activity was fully removed, and neurogenic components were unaffected during waveform alteration.”

 16. (page 12, fourth paragraph) “ *(…) (1) reliably produce signals that are, exclusively or mainly, EEG or MEG (…)”*. Please clarify and elaborate on this claim.

 -- We agree with your comment that this sentence is unclear. This paragraph has been modified in the revision and now reads as follows: “One explanation for AR2’s ability to isolate myogenic from neurogenic activity may be related to the respective dipole generators of each. ICA produces independent components that may resemble single equivalent dipoles ^14^. Presumably, networks of myocytes exhibit shorter distance connectivity then networks of neurons that produce beta and gamma oscillations, and thus the two generators can be distinguished on the basis of the focality ^17^ of the independent components topography.”  

**References**

 1. Chaumon M, Bishop DV, Busch NA: A practical guide to the selection of independent components of the electroencephalogram for artifact correction. *J Neurosci Methods*. 2015; **250**: 47-63 PubMed Abstract | Publisher Full Text
